# Isolation and characterization of Uropathogenic *Escherichia coli* (UPEC) from red panda (*Ailurus fulgens*)

**DOI:** 10.1186/s12917-020-02624-9

**Published:** 2020-10-27

**Authors:** Songrui Liu, Yunli Li, Chanjuan Yue, Dongsheng Zhang, Xiaoyan Su, Xia Yan, Kuixing Yang, Xin Chen, Guifu Zhuo, Tong Cai, Jiangfeng Liu, Xi Peng, Rong Hou

**Affiliations:** 1 Chengdu Research Base of Giant Panda Breeding, Chengdu, 610081 Sichuan China; 2 Sichuan Key Laboratory of Conservation Biology for Endangered Wildlife, Chengdu, 610081 Sichuan China; 3 Sichuan Academy of Giant Panda, Chengdu, 610081 Sichuan China; 4 Key Laboratory of Southwest China Wildlife Resources Conservation (Ministry of Education), Nanchong, 637009 Sichuan China; 5 College of Life Science, China West Normal University, Nanchong, 637009 Sichuan China

**Keywords:** Red panda, Uropathogenic *Escherichia coli*, Pathological diagnosis

## Abstract

**Background:**

Disease prevention and control is a significant part in the ex-situ conservation of the endangered red panda *(Ailurus fulgens),* being bacterial infection is one of the most important health threats to the captive population. To date, studies about the infection caused by *Escherichia coli* in the red panda are scarce. This study was conducted to determine the cause of death of a captive red panda through clinical symptoms, complete blood count, biochemical analysis, pathological diagnosis and bacterial whole genome sequencing.

**Case presentation:**

The following report describes a case of a 1.5 year old captive red panda (*Ailurus fulgens*) that was found lethargic and anorectic. She was moved to the quarantine area for daily treatment with 50 mg of Cefpodoxime Proxetil. During the three-day treatment, she did not eat or defecate, and then died. Clinical hematology revealed the values of neutrophils, alanine aminotransferase (ALT), aspartate aminotransferase (AST) and blood urea nitrogen (BUN) were significantly higher. Histological analysis demonstrated major pathological damage in the kidneys, liver and lungs, characterized by hyperemia, parenchymal cell degeneration and necrosis and inflammatory cell infiltration which were predominantly neutrophilic. A bacterial strain confirmed as *Escherichia coli* was isolated post mortem. Whole genome sequencing of the *E. coli* showed the complete genome size was 4.99 Mbp. *PapA*, *PapC*, *OmpA*, *OmpU* and other virulence factors which specific to Uropathogenic *Escherichia coli* (UPEC) were found in the isolate. Among the virulence factors, P pili, type I pili and related factors of the iron uptake system were associated with nephrotoxicity.

**Conclusion:**

The red panda died of bacterial infection caused by an uropathogenic strain of *Escherichia coli*. The pathogenic mechanisms of the strain are closely related to the expression of specific virulence genes.

**Supplementary Information:**

**Supplementary information** accompanies this paper at 10.1186/s12917-020-02624-9.

## Background

*Escherichia coli* (*E. coli*) is a common species of bacteria distributed in the digestive tract of humans and animals, but most of the strains have no pathogenic affect. Some strains may acquire virulence factors, turning into pathogenic bacteria, causing infections in animals and humans [1]. *E. coli* infection occurs in poultry and livestock, as well as in wild animals such as snub-nosed monkeys and giant pandas [2–5]. Uropathogenic *Escherichia coli* (UPEC) can induce urinary tract infection through entering the urethra, then causing pyelonephritis, cystitis and urethritis [6–8]. If the bacterial infections cannot be controlled in time, renal function damage may occur and pose a serious threat to health and life.

The red panda *(Ailurus fulgens)* has been classified as an endangered species by the International Union for the Conservation of Nature (IUCN) as its wild population is estimated at less than 10,000 mature individuals, and its survival in the wild is threatened by deforestation, loss of habitat and fragmentation of the existing wild populations [9]. Disease prevention and control is a significant part in the ex-situ conservation of the red panda, being bacterial infection is one of the most important health threats to the captive population. To date, studies about the infection caused by *Escherichia coli* in the red panda are scarce. Analysis of post-mortem reports is an important tool in increasing our understanding of the red panda in captivity and improving our husbandry and management procedures for this species [10]. Due to its special ecological status, prevention and treatment of *E. coli* infection in the red panda needs high attention.

This study was conducted to determine the cause of death, etiology and pathogenesis in a red panda. The cause of death was identified through clinical symptoms, complete blood count, biochemical analysis, gross and histopathological diagnosis. A strain of *E. coli* was isolated and identified, and bacterial whole genome sequencing was used to explore the pathogenic mechanism of the bacteria. The results provide a scientific basis for the diagnosis and clinical treatments of the bacterial infection in the red panda.

## Case presentation

### Clinical history

A 1.5 year old captive female red panda (*Ailurus fulgens*) was found to be lethargic and anorectic at Panda Valley, the Chengdu Field Research Center for Giant Pandas in Dujiangyan, China. Oral and nasal secretions were collected for the search of Canine Distemper Virus (CDV) and Canine Parvovirus (CPV) using AsanEsay Test (ASAN PHARM. Co., LTD), and the results were negative. It was anaesthetized with 21 mg Zoletil (intramuscular injection) at a dose of 3 mg/kg body weight, and treated with an intravenous injection of 350 mg Sulperazon (Cefoperazone Sodium and Sulbactam Sodium) in 150 mL 0.9% NaCl. The complete blood count and biochemical analysis results are shown in Additional file 1. Compared with a previous report on blood counts and biochemical parameters of 28 healthy red pandas, the values of neutrophils, alanine aminotransferase (ALT), aspartate aminotransferase (AST) and blood urea nitrogen (BUN) found in this study were significantly higher while remaining parameters were normal [11]. She was moved to the quarantine area of the Chengdu Research Base of Giant Panda Breeding for daily treatment of oral Cefpodoxime Proxetil 50 mg. During the three-day treatment, she did not eat or defecate, and then died.

### Necropsy

During necropsy, the red panda’s skin and fur was intact, and no traumatic injuries were observed. Both kidneys were swollen with several white pinpoint spots on the cutting surface, with white cord-like streaks in the medulla. The liver was icteric, with soft and brittle texture, and few dark red ecchymoses on the cutting surface. The lungs were dark red with multifocal plaques of black-red hemorrhage. The spleen was swollen and dark red with a small number of black-red plaques. The heart and intestines were intact, with no obvious lesions.

### Histopathology

**Fig. 1:**
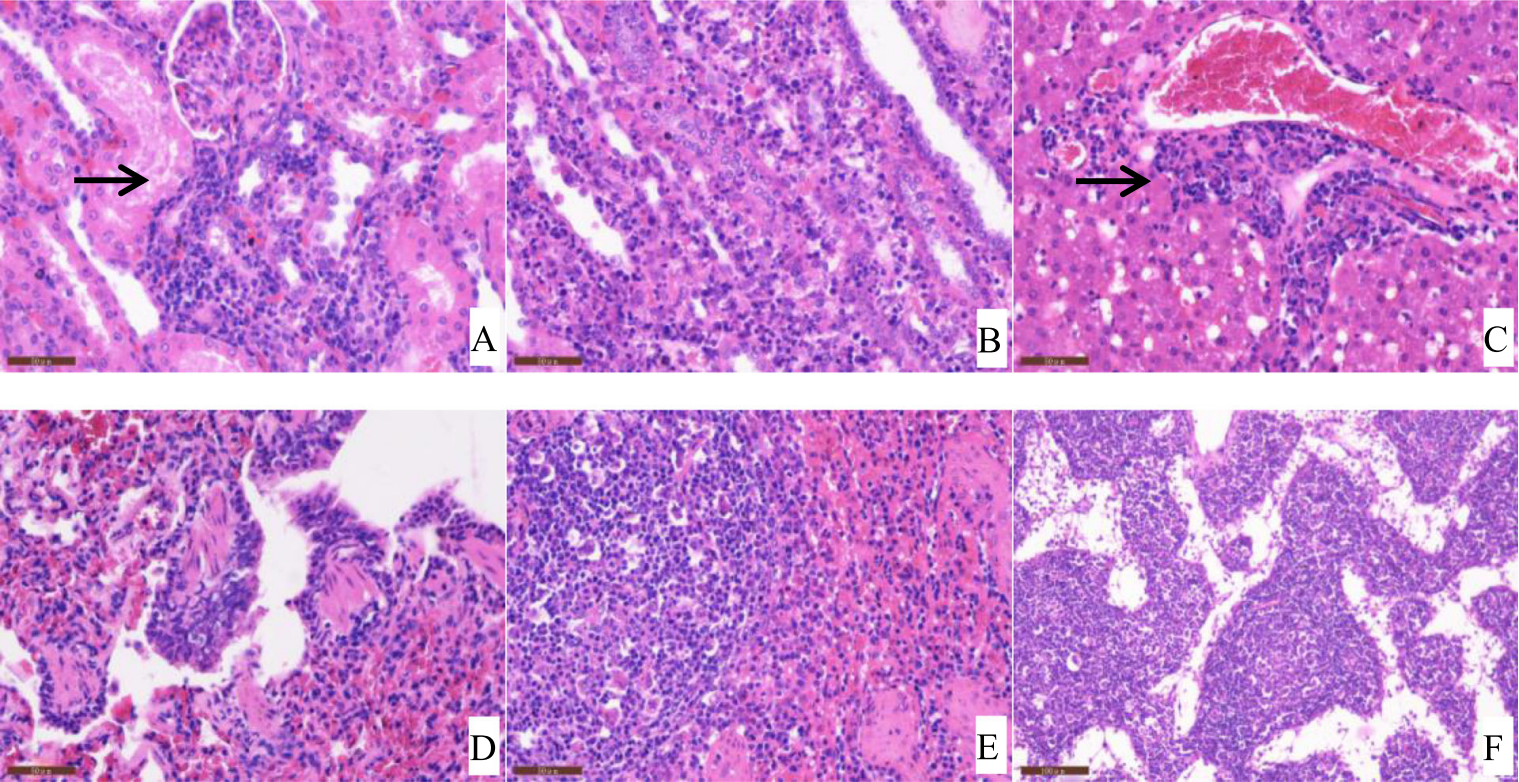
Histopathological findings with UPEC infection. **a**: Glomerular congestion, epithelial cell necrosis in small renal tubules, and focal infiltration of inflammatory neutrophils and fewer lymphocytes (*arrow*). **b**: Cord-like infiltration of lymphocytes and plasma cells in the renal medullary interstitium. **c**: Mild hepatic congestion, perivascular infiltration of lymphocytes and plasma cells (*arrow*), and hepatocyte vacuolar degeneration. **d**: Thickening, hyperemia of the pulmonary alveolar wall and infiltration of lymphocytes and plasma cells. **e**: Congestion and hemorrhage in splenic red pulp. **f**: Expansion of the mesenteric lymph node sinuses by infiltration of lymphocytes and plasma cells. H&E, bar = 50 μm

Specimens, including the heart, liver, spleen, lung, kidney, gastrointestinal tract and other tissues were fixed in 4% paraformaldehyde, and routinely processed in paraffin. Sections (5 μm) were stained with haematoxylin and eosin Y (H&E) and evaluated for histopathological changes under the microscope (Leica DM4B optics). Histopathological analysis revealed significant glomerular congestion in the renal cortex, necrosis of renal tubular epithelial cells, collapse of renal tubular lumens and interstitial infiltration of neutrophils, lymphocytes, plasma cells and macrophages (Fig. 1a). Cord-like interstitial inflammatory lesions were noted in the renal medulla regions, as normal collecting duct structures were replaced by extensive inflammation (Fig. 1b). In the liver, there was vesicular degeneration or steatosis of hepatocytes, mild congestion, and perivascular inflammatory cell (neutrophils, macrophages and lymphocytes) infiltration (Fig. 1c). In the lung, alveolar walls were thickened by lymphoplasmacytic infiltration and congestion. Some alveolar spaces collapsed, while others were dilated due to compensatory emphysema (Fig. 1d). Expanded lymphoid follicular germinal centers showed increased splenic white pulp, while red pulp showed congestion, hemorrhage and inflammation with nuclear debris (Fig. 1e). Mesenteric lymph node sinuses were dilated with increased macrophages and lymphocytes (Fig. 1f). Other tissues (heart, pancreas, stomach, digestive tract) did not show any pathological lesion.

### Bacterial isolation and molecular identification

**Fig. 2:**
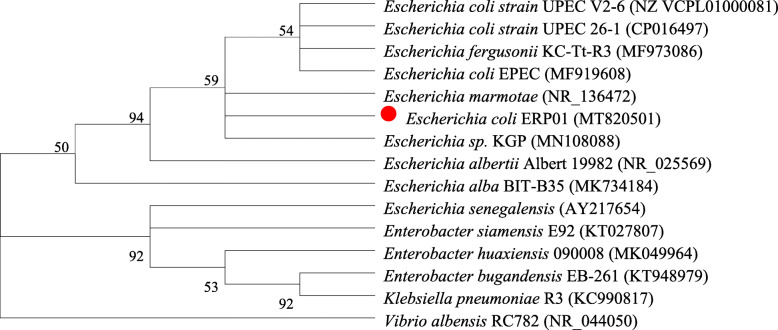
Phylogenetic analysis of the isolated strain and close related species using the Neighbor-Joining method. Bootstrap values (expressed as a percentage of 1000 replications) > 50% are given at the branching points. *Vibrio albensis* strain RC782 (NR_044050) was used as outgroup

Strains were isolated from the kidney, liver and lung samples. Genomic DNA was individually extracted from the isolates using the TIANamp Bacteria DNA kit according to the manufacturer’s instructions (Tiangen Biotech Co., Ltd., Beijing, China). The 16S rRNA gene was amplified by PCR performed in a T100™ Thermal Cycler (Bio-rad, USA). The amplified 1500 bp 16S rRNA genes of the isolated strains shared the highest identity (99.45%) with that of *E. coli* (GenBank: LC050175.1), and the phylogenetic analysis demonstrated that the isolated strain and *E. coli* strains have high phylogenetic relatedness using the Neighbor-Joining method (Fig. 2). The sequence of the isolated strain was submitted to GenBank under the accession number MT820501.

### Whole genome sequencing

Genomic DNA was extracted using a sample of high-quality, high-molecular-weight DNA prepared using the TIANamp Bacteria DNA kit according to the manufacturer’s instructions (Tiangen Biotech Co., Ltd., Beijing, China). The sample was sent to an external laboratory for whole genome sequencing using the PacBio RS II platform (completed by Biomarker Technologies Co, LTD.). The complete genome size of the *E. coli* chromosome isolated from the red panda was 4,990,420 bp (GenBank: PRJNA668015). BLAST alignment of the predicted protein sequence with the Antibiotic Resistance Gene Database (ARDB) showed that the strain contained 20 resistance genes (Additional file 2) including *acra*, *acrb*, *mdte*, *mdtf*, *mdtn*, *mdto*, *mdtp*, *tolc*, *arna*, *baca*, *bcr*, *bl1_ec*, *emre*, *ksga*, *macb*, *mdfa*, *mdtg, mdth*, *mdtk* and *mdtl*. These genes can mediate resistance to aminoglycosides, β-lactams, macrolides and other antibiotics through resistance-nodulation-cell division transporter system, multidrug resistance efflux pump and other ways. BLAST alignment with the Virulence Factor Database (VFDB) showed that the measured *E. coli* contained 713 virulence factors (Additional file 3), including outer membrane protein, flagella, P pili, S pili, type I pili, cytotoxic necrosis factor, and hemolysin. In addition, factors related to the iron uptake system and other types of systems were identified. Among these virulence factors, *PapA*, *PapC*, *OmpA*, *OmpU* and other virulence factors were specific to Uropathogenic *Escherichia coli* (UPEC) [8], thus confirming its identity.

## Discussion and conclusions

Understanding and addressing disease threats in the ex-situ population of the red panda is crucial to the conservation of in-situ populations of this species in China. In this study, blood analysis revealed leukocytosis with increased ALT, AST and BUN, suggesting that bacterial infection caused liver and kidney damage, followed by death [12–14]. Histopathological findings revealed major pathological damage in the kidneys, liver and lungs, characterized by hyperemia, parenchymal cell degeneration and necrosis, inflammatory cell infiltration which was predominantly neutrophilic. These findings are consistent with the results of the complete blood count and biochemical analysis. Researchers have reported acute pyelonephritis caused by UPEC in the mouse model, with renal histopathological lesions including infiltration of inflammatory cells (predominantly neutrophils) in renal pelvis submucosa, renal interstitial dilatation, partial renal tubular epithelial cell necrosis and shedding [15–17]. These were similar to the pathological manifestations of the kidney in this red panda. We conclude that UPEC induced pyelonephritis in this red panda, through retrograde infection from the urethra, then lead to septicemia.

Although copy numbers of *acra*、*tolc*、and *emre* genes (included in multi-drug efflux systems which mediate the aminoglycoside、polymyxin and beta_lactam antibiotic resistance) were detected, this *E. coli* isolate was not sensitive to Gentamycin and Cefoperazone. As far as the clinical treatment of the red panda, when symptoms were first noted, Sulperazon (Cefoperazone Sodium and Sulbactam Sodium) and Gentamicin were used, but there was no obvious curative effect. Current increasing usage of the clinical antibacterial drugs forces antibiotic resistance to occur in both pathogenic and zoonotic bacteria in animals [18]. Previous research showed that the antibiotic resistance strains can make treatment of bacterial infections more difficult, leading an overall increase in transmission, morbidity and mortality [19, 20]. These results suggest that effective treatment should be based on the results of antimicrobial susceptibility tests and clinical efficacy in the control of bacterial infections in clinical practice.

Previous researchers found that 80% of *E. coli* containing P pili may cause pyelonephritis [21]. In our study, the whole genome sequencing results found high express of P pili and typeIpili genes. The P pili receptors exist on renal epithelia, while type I pili mediates the biofilm formation and colonization of the bacteria in the renal epithelia [8], which makes the strain more nephrotoxic. In addition, the virulence factors associated with the iron uptake system are also high in this case. The presence of the iron uptake system enhances the pathogenicity of the bacteria, contributing to the bacterial survival through the host’s heme and ferritin [22].

Based on the results obtained, we can conclude the red panda died of septicemic bacterial infection and the isolated bacterial strain was identified as UPEC. Since this is the first report of UPEC strain isolation in the red panda, further research is needed for a better understanding of the epidemiology, susceptibility and antibiotic resistance of these types of *E. coli* strains in the red panda.

## Supplementary Information

**Additional file 1.** Complete blood count and biochemical analysis for the red panda.

**Additional file 2. **Antibiotic resistance protein of the *E. coli* chromosome isolated from the red panda.

**Additional file 3. **Virulence factor protein of the *E. coli* chromosome isolated from the red panda.

## Data Availability

All the nucleotide sequences are deposited in GenBank and available under accession numbers MT820501 and PRJNA668015. Other data generated or analyzed during this study are included in additional files.

## Abbreviations

UPEC: Uropathogenic *Escherichia coli*
IUCN: International Union for Conservation of Nature
CBC: Complete blood count
ARDB: Antibiotic resistance gene database
VFDB: Virulence factor database
CDV: Canine distemper virus
CPV: Canine parvovirus
WBC: White blood cells
NEU: Neutrophils
RBC: Red blood cells
HGB: Hemoglobin
MCH: Mean corpuscular hemoglobin
MCHC: Mean corpuscular hemoglobin concentration
PLT: Platelets
TP: Total protein
TB: Total bilirubin
ALT: Alanine aminotransferase
AST: Aspartate aminotransferase
CK: Creatine kinase
TC: Total cholesterol
BUN: Blood urea nitrogen
CR: Creatinine
UA: Uric acid
GLU: Glucose
BHI: Brain heart infusion
IACUC: Institutional Animal Care and Use Committee
