# Supplementary material for: Isolation and characterization of Uropathogenic Escherichia coli (UPEC) from red panda (Ailurus fulgens)
Source: BMC Vet Res. 2020 Oct 27;16:404. doi: 10.1186/s12917-020-02624-9 (PMC7590469; doi:10.1186/s12917-020-02624-9)
Supplement: Supplementary file 1 — Additional file 1. Complete blood count and biochemical analysis for the red panda. [file 12917_2020_2624_MOESM1_ESM.docx]

Complete blood count and biochemical analysis for the red panda

| Projects | Units | Value | Reference range [11] |
| --- | --- | --- | --- |
| WBC | ×10^9^/L | 9.66 | 7.24-15.76 |
| RBC | ×10^9^//L | 6.48 | 6.35-10.8 |
| Neu | ×10^9^/L | 6.97 | 2.36-10.4 |
| Neu (%) | - | 72.2 | 16.4-48 |
| HGB | g/L | 91 | 87-149 |
| MCH | Pg | 14.1 | 12.9-14.55 |
| MCHC | g/L | 342 | 310-367 |
| PLT | - | 571 | 352-738 |
| TP | g/L | 83.1 | 74-104 |
| TB | μmol/L | 2.37 | 0.50-5.60 |
| ALT | U/L | 1127 | 22-154 |
| AST | U/L | 482 | 51-137 |
| CK | U/L | 485 | 113-608 |
| TC | mmol/L | 6.22 | 5.81-9.98 |
| BUN | mmol/L | 13.64 | 4.00-10.20 |
| Cr | μmol/L | 110 | 56-112 |
| UA | μmol/L | 42.3 | 47-137 |
| GLU | mmol/L | 5.01 | 4.00-13.22 |
